# Identifying genes required for respiratory growth of fission yeast

**DOI:** 10.12688/wellcomeopenres.9992.1

**Published:** 2016-11-15

**Authors:** Michal Malecki, Jürg Bähler

**Affiliations:** 1 Research Department of Genetics, Evolution & Environment and Institute of Healthy Ageing, University College London, London, UK; 2 Department of Genetics and Biotechnology, Faculty of Biology, University of Warsaw, Warsaw, Poland

**Keywords:** Respiration, fission yeast, energy metabolism, mitochondria, glycerol, fermentation, functional profiling, mutant screen

## Abstract

We have used both auxotroph and prototroph versions of the latest deletion-mutant library to identify genes required for respiratory growth on solid glycerol medium in fission yeast. This data set complements and enhances our recent study on functional and regulatory aspects of energy metabolism by providing additional proteins that are involved in respiration. Most proteins identified in this mutant screen have not been implicated in respiration in budding yeast. We also provide a protocol to generate a prototrophic mutant library, and data on technical and biological reproducibility of colony-based high-throughput screens.

## Introduction

Energy metabolism is fundamental for cell growth and function, and cells need to tune metabolic pathways to optimize their physiology in response to different nutrient or physiological conditions ^1^. Metabolism in yeast can be manipulated by the growth medium: on glucose yeast cells proliferate by fermentation, while on glycerol they shift towards respiration. Much of our fundamental knowledge on energy metabolism stems from research with *Saccharomyces cerevisiae*, including systematic screens for genes involved in cellular respiration ^2, 3^, while *Schizosaccharomyces pombe* provides a potent complementary model system ^4^. We have recently applied large-scale functional and regulatory analyses on energy metabolism in fission yeast ^5^. The data presented here provide additional, complementary insights into genes that are required for respiration.

Here, we screened the *S. pombe* deletion collection ^6^ for mutants affecting growth on solid media containing glycerol as a main carbon source. Glycerol is a non-fermentable carbon source, which forces cells to gain energy by respiration rather than fermentation. We compared respiratory growth on glycerol to fermentative growth on glucose (control) to identify mutants that show defects in respiration. This screen differed in three respects from screens we previously reported ^5^: 1) we used the latest version 5 of the gene-deletion library, which features over 400 more mutants (3420 vs 3003) and corrections of erroneous deletions; 2) we generated a prototrophic library of this latest version using an improved protocol that minimizes contamination by wild-type cells and optimizes library quality; 3) we determined colony sizes at densities of only 384 colonies per plate (instead of 1536 colonies), since larger colonies provide a higher dynamic range and sensitivity that outperforms data from higher density screens despite the reduced number of technical repeats; and 4) we applied a different data analysis and only used glycerol as a non-fermentable carbon source, while the previous study also measured respiratory growth on galactose ^5^.

## Materials and methods

### Yeast media

YES: 5g/L yeast extract, 30g/L glucose, 0.25g/L adenine, histidine, leucine, lysine, uracil (Formedium PMCUCL1000)

EMM agar (Formedium PMD0100)

ME agar (Formedium PCM 0810)

YE glucose: 5g/L yeast extract (Bacto 212750), 30g/L glucose (Fisher G/0500/53)

YE glycerol: 5g/L yeast extract (Bacto 212750), 30ml/L glycerol Fisher BP229-1), 1g/L glucose (Fisher G/0500/53)

For solid media, 2% agar (VWR 20767.298) was added.

### Auxotroph deletion library

The auxotroph haploid deletion library version 5.0 was obtained from Bioneer ( http://www.bioneer.com/). The basic genotype is *h+ ade6-M210 (or ade6-M216) ura4-D18 leu1-32*. Each strain contains a different non-essential gene deleted with the kanamycin cassette ^6^. This library contains 3420 strains.

### Prototroph deletion library

The prototroph deletion library was prepared by backcrossing the auxotroph deletion library to a *h ^-^ 972* strain with its H1 box deleted with a hygromycin cassette ( *h ^-^ 972 H1::hyg ^r^*; strain created in this work). The cassette was amplified on the template of the pFA6a family plasmid ^7^ using the following primers (Life Technologies) (Left: AAAGTAATACATGGATTTTACTGCCCTGATTCTATCGAAATATGCTGTTTTTTTTATTCGTTTTTATTTATTTTCAATAACGGATCCCCGGGTTAATTAA; Right: GGGAAGGGGAAGGTAGAAGGGCGCACACAAAAAGGGAAAATTGGAGGGAGAATGAATGACACGAACAGCATAATTGGAAAGAATTCGAGCTCGTTTAAAC). Replacing the H1 box with hygromycin allows to select stable *h ^-^* mating types in the progeny; this mating-type selection prevents mating during the screen, and enables the use of this prototroph collection for synthetic genetic array assays.

### Prototroph deletion library preparation

The auxotroph deletion library was arrayed onto 9 PlusPlates (Singer Instruments) with solid YES medium, at 384 colonies per plate using the RoToR HDA robot (Singer Instruments), and grown for 2 days at 32°C. The prototroph strain ( *h ^-^ 972 H1:: hyg ^r^*) was grown in liquid YES medium overnight and subsequently arrayed onto 3 PlusPlates with YES medium at 384 colonies per plate and grown for 3 days at 32°C. The freshly grown prototroph strain was then mixed with the freshly grown auxotroph deletion collection on PlusPlates with solid ME medium, using the RoToR HDA robot mate protocol. Each plate of wild-type strains was used to mix with 3 plates of the auxotroph collection. Subsequently, the 9 plates obtained were wrapped in cling film and stored at 25°C for 3 days to facilitate mating and sporulation.

After incubation, the plates were moved into a well humidified 45°C incubator for another 3 days. This step is designed to kill parental strains and diploids, because only spores are resistant to this high temperature. Next, using the RoToR HDA robot, spores were copied onto PlusPlates with YES medium and grown for 2 days at 32°C to allow spore germination before the selection steps. To improve efficiency, the selection was performed first in liquid medium. Cells were transferred from PlusPlates with solid YES medium to 384-well plates filled with 60µl/well of YES containing hygromycin and kanamycin (0.1mg/ml each) and kept overnight at 32°C. Subsequently, cells were transferred onto agar PlusPlates with YES supplemented with hygromycin and kanamycin (0.1 mg/ml each) and grown for two days at 32°C. For the second selection step, cells were transferred from YES plus hygromycin and kanamycin plates onto a 384-well plate filled with 60µl/well of EMM medium and grown overnight at 32°C. Cells were then copied onto PlusPlates with solid EMM medium and grown for 2 days at 32°C. Subsequently, cells were transferred onto PlusPlates with YES supplemented with hygromycin and kanamycin and grown for two days. The resulting prototroph library was then transferred onto 384-well plates with 60µl of YES with 20% glycerol and stored at -80°C.

### Screening procedure

**Figure 1.**
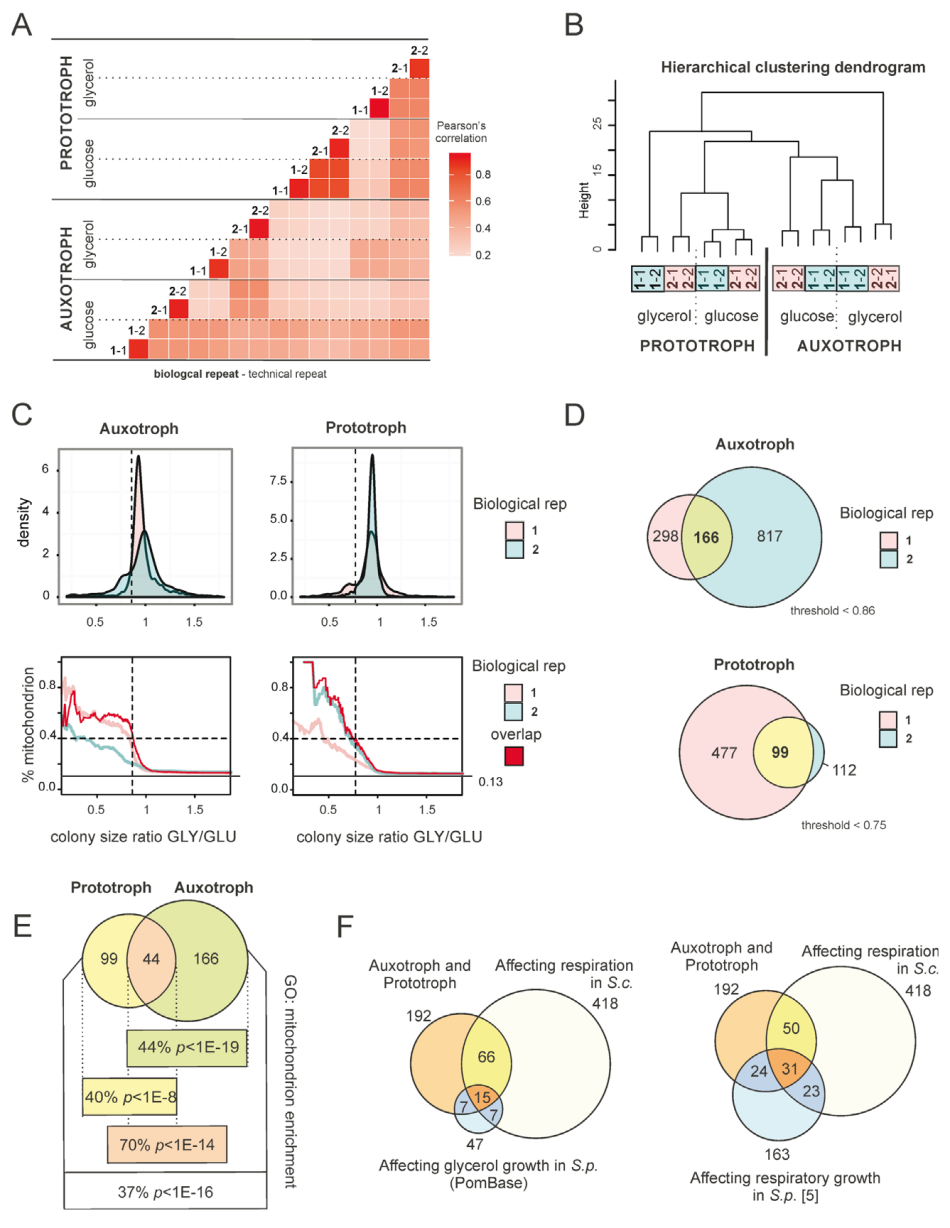
Validation of screen results. ( **A**) Relationships between all 16 data sets, consisting of biological and technical repeats on fermentative (glucose) and respiratory (glycerol) media for both auxotroph and prototroph libraries as indicated. Pearson’s correlations between data sets are visualised on the heat map using the *ggplot2* R package v.2.1.0 ( http://ggplot2.org/; ggcorr function, method “pairwise”, “pearson”). The biological repeats are indicated in bold numbers, followed by numbers for technical repeats. ( **B**) Hierarchical clustering of all 16 data sets using the *dist* (method “Euclidean”) and *hclust* (method “ward.D”) R functions. Data sets are labelled as in ( **A**), with biological repeats colour-coded as in ( **C**) and ( **D**). ( **C**) Upper graphs:Distributions of colony size ratios (growth on glycerol/growth on glucose). Data from technical repeats for glucose and glycerol plates are averaged and colony size ratios calculated for each biological repeat of both prototroph and auxotroph libraries. The vertical dashed lines indicate the chosen thresholds for gene lists in the lower graphs. Lower graphs: Functional enrichments between biological repeats. The percentages of genes with the Gene Ontology (GO) term ‘mitochondrion’ (for each biological repeat and the overlap list, see colour legend) are plotted as a function of different colony-size ratios for both auxotroph (left) and prototroph (right) libraries. The vertical dashed lines indicate the thresholds chosen to generate gene lists that show ≥40% enrichment for the GO term ‘mitochondrion’ (horizontal dashed lines). The solid horizontal line indicates the proportion of genes associated with the GO term ‘mitochondrion’ among all genes (~13%). ( **D**) Overlaps between biological repeats. The chosen colony-size ratio thresholds ( **C**) were used to generate gene lists for both biological repeats and genetic backgrounds. Venn diagrams with numbers of genes whose mutants showed colony-size ratios values lower than the thresholds. The overlapping genes were used for further analyses in ( **E**) and ( **F**). ( **E**) Gene lists obtained from the chosen colony-size ratio thresholds ( **C**) for auxotroph and prototroph libraries and their overlap were tested for enrichment of genes associated with the GO ‘mitochondrion’ term using AnGeLi ^9^. The percentages of mitochondrial genes and the corrected *p* values for the enrichment of mitochondrial genes is provided for each gene list, colour-coded as in the Venn diagram. ( **F**) Gene lists obtained from the chosen colony size ratio thresholds ( **C**) for auxotroph and prototroph libraries were combined (Auxotroph and Prototroph), and compared with three other relevant gene lists: 1) genes which have previously been annotated in PomBase to affect *S. pombe (S.p)* growth on glycerol (left Venn diagram); 2) genes which we have independently identified ^5^ to affect respiratory growth (right Venn diagram); and 3) genes affecting respiratory growth in *S. cerevisiae* ( *S.c.*), obtained from 3 phenotype categories in the *Saccharomyces* genome database ( www.yeastgenome.org): respiratory growth absent, decreased or decreased rate. All the lists were limited to genes present in the *S. pombe* deletion library (some respiratory genes are essential in *S. pombe,* but not in *S. cerevisiae*) and showing orthologues in both yeasts, based on the manually curated list in PomBase ^10^.

For each biological repeat, the deletion library (prototroph or auxotroph) was freshly plated from -80°C glycerol stocks onto PlusPlates with solid YES medium at 384 colonies per plate. After 3 days incubation at 32°C, libraries were copied onto YES plates with kanamycin, and incubated again for 2 days, then were copied again onto two sets of PlusPlates with solid YES medium and incubated for two more days. These plates were used as an initial template for the screen and treated as technical repeats. The library from each set of YES plates was copied onto PlusPlates with YE glucose or YE glycerol medium and grown for at least 2 days at 32°C. Cells grew more slowly on YE glycerol and were left for up to four days. After incubation, pictures of the plates were taken using a Canon camera (PC1305) and MultiDoc-It imaging system (UVP). In total, two technical repeats of the screen were obtained for each of the two biological repeats carried out, with both the auxotroph and the prototroph libraries, resulting in four sets of colonies grown on glycerol medium and four sets of colonies grown on glucose medium to be recorded for each library ( Figure 1A and B).

### Data treatment

Quantitation of colony sizes was performed with the *gitter* R package (v. 1.1.1) ( http://omarwagih.github.io/gitter/) ^8^. For normalisation, we used an in-house tool (Townsend StJ., Rallis C., Bähler J., unpublished data). In brief, absent colonies were identified and excluded from the normalisation process; for each plate, colony sizes were normalised to the plate median, corrected for row- or column-specific artefacts (i.e. edge effects). A median filter was applied to identify and correct for local spatial variations. Initial data analysis revealed some noise, although correlations between data sets were all positive, with the strongest correlations being obtained for the technical repeats and with weaker correlations between biological repeats grown on the same carbon source ( Figure 1A). Similarly, clustering analysis showed that data are most similar among technical repeats ( Figure 1B). Due to the high variability between biological repeats, we decided not to average values from the 4 repeats for each carbon source. First, we only averaged data from the corresponding technical repeats, and filtered out colonies showing high variability between repeats (standard error >0.4), being absent in one of the repeats, or with very small colony sizes on glucose (normalised median size <0.12). Second, we separately calculated for each biological repeat the colony size ratios between glycerol and glucose.

## Dataset validation

We obtained 16 sets of colony size data from the screen: 8 sets each for the auxotroph and prototroph mutant library backgrounds, consisting of 2 independent biological repeats each with colonies grown on either YE glucose or YE glycerol media, with each biological repeat consisting of two technical repeats. The data from technical repeats strongly correlated, while the data from biological repeats showed much weaker correlations ( Figure 1A and B). There was also a substantial difference between most data based on the auxotroph library and those based on the prototroph library ( Figure 1B). The distributions of colony-size ratios (growth on glycerol/growth on glucose) also differed substantially between the biological repeats ( Figure 1C, upper graphs).

We analysed the top-100 screen hits showing the lowest colony-size ratios for each of the 4 biological repeats for functional enrichments within the Gene Ontology (GO; http://geneontology.org/) ‘Cellular Component’ category, using the AnGeLi tool ( www.bahlerlab.info/AnGeLi) ^9^ with the deletion library genes as a background list. Since the screen was designed to detect respiratory-deficient mutants, we expected an enrichment in genes encoding mitochondrially localised proteins. This analysis revealed the expected enrichments in the GO term ‘mitochondrion’ (GO: 0005739 PomBase, www.pombase.org ^10^), which reached 50% for biological repeat 1 (p <1.1E-15) and 40% for biological repeat 2 (p <1.2E-8) for the auxotroph library, and 33% for biological repeat 1 (p <5E-4) and 37% for biological repeat 2 (p <5.5E-6) for the prototroph library. These strong enrichments in the key expected GO term indicate that the screen produced biologically relevant results. We then analysed the overlap of genes obtained from the 2 biological repeats for both the auxotroph and prototroph libraries using a threshold value. To choose the optimal threshold for each library, we plotted the enrichments in genes with the GO term ‘mitochondrion’ as a function of increasing thresholds for the biological repeats and overlap ( Figure 1C, lower graphs). The thresholds were set such that the overlap between the biological repeats showed ≥40% enrichment for the GO term ‘mitochondrion’. For the auxotroph library, a threshold of <0.86 for the colony-size ratio (growth on glycerol/growth on glucose) gave an overlap of 166 genes between the biological repeats, which showed 44% enrichment in genes associated with the GO term ‘mitochondrion’ ( Figure 1D and E). For the prototroph library, a threshold of <0.75 for the colony-size ratio gave an overlap of 99 genes, which showed 40% enrichment in genes associated with the GO term ‘mitochondrion’ ( Figure 1D and E).

We then merged the results obtained from both genetic backgrounds (auxotroph and prototroph). The GO term ‘mitochondrion’ showed an enrichment of 37% in the merged list ( Figure 1E). Substantial overlaps were also evident with independently identified genes affecting *S. pombe* growth on glycerol (FYPO: 0001934 PomBase ^11^), as well as with genes resulting from our previous screen using an older version of the Bioneer deletion library (version 2.0; http://www.bioneer.com/) and a different prototroph library ^5^ ( Figure 1F). We also compared our screen hits with orthologous genes that affect respiratory growth in budding yeast ( *Saccharomyces* genome database; www.yeastgenome.org) *,* where the genes resulting from the new screen showed a stronger overlap than the genes from our previous screen ( Figure 1F).

## Conclusions

As we have shown before ^5^, the genetic background of the deletion library can strongly affect the screen results. The commonly used auxotroph mutant background has shortcomings for investigating energy metabolism in particular, yet also provides complementary insights. We find that the mutant library in the prototroph background shows less variation in growth as measured by colony sizes, which likely reflects the absence of genetic interactions from the three auxotrophic mutants. We also report quite large variations among independently repeated screens, which highlights the importance to perform biological repeats. The genes implicated in respiration in the previous screen ^5^ and in the new screen reported here compare as follows: 149 genes have only been identified in the previous screen, 55 genes have been identified in both screens, and 166 genes have only been identified in the new screen. These substantial differences not only reflect the differences in the mutant library and methods (see above), but also the large variations in respiratory-deficient phenotypes, as also observed in *S. cerevisiae* ^2^. This variation and experimental noise may be even higher in *S. pombe* for the following reasons: 1) *S. pombe* depends on respiration to a greater extent than *S. cerevisiae* ^12^, and respiratory mutants may therefore affect growth also in fermentative media, thus diminishing growth differences between respiratory and fermentative conditions; and 2) *S. pombe* generally grows very poorly on glycerol, which reduces the sensitivity to record differences in colony sizes. The data reported here provide an additional resource of respiratory genes, which complements and enhances data from the previous screens.

Taken together, the partial overlaps between data obtained in different screens, as well as the variability of the data from different biological repeats and strain backgrounds, suggests a high plasticity of the respiratory phenotype. A similar phenomenon has been noticed in budding yeast ^2^. Thus, merging data from different high-throughput screens provide complementary information and together increase the sensitivity to systematically identified genes relevant for respiratory growth.

## Data availability

Raw data are deposited in the Open Science Framework (DOI: http://dx.doi.org/10.17605/OSF.IO/VCHE3 ^13^; https://osf.io/vche3/). Data from this and the previous screen ^5^ will be also be available on our website ( www.bahlerlab.info/resources).
