# Supplementary material for: Identifying genes required for respiratory growth of fission yeast
Source: Wellcome Open Res. 2016 Nov 15;1:12. [Version 1] doi: 10.12688/wellcomeopenres.9992.1 (PMC5133385; doi:10.12688/wellcomeopenres.9992.1)
Supplement: Peer review history

# Peer review: Referee response for version 1

**DOI:** 10.21956/wellcomeopenres.10768.r17616

**Published:** 2016-11-28

**Authors:** Makoto Kawamukai

**Affiliations:** 1 Department of Life Science and Biotechnology, Faculty of Life and Environmental Science, Shimane University, Matsue, Japan

The work done by Malecki and Baehler is the screening of the S. pombe mutants that did not grow well on the yeast extract medium containing mainly glycerol comparing with glucose. They used auxotrophic and prototrophic mutants and repeated experiments twice. Statistical analysis was conducted. The data presented here is sound and is useful. A couple things I am concerned.

1. Variation of two independent experiments looks high. It is partly because they judged by only plate works. To be truly respiration defective, the authors are better to measure oxygen consumption of the mutants. That is the definition of respiration deficiency.
2. Did the authors find out the mutants that are required for glycerol metabolism but not relevant to respiration? Did the authors omit these mutants from the list of respiration defective mutants. I could not reach the list of the mutants the authors selected.
3. We are also using a deletion mutant library of Bioneer Corp. It still contains the strains that are not deleted properly or apparently possess extragenic unknown mutation(s). Several must-be-respiration defective mutants such as coenzyme Q less mutants we are studying ^1^ are not included in the Bioneer library. Without re-checking by own-self, it still needs to be careful for interpretation using this library.

I have read this submission. I believe that I have an appropriate level of expertise to confirm that it is of an acceptable scientific standard.

## References

[1] HayashiKOgiyamaYYokomiKNakagawaTKainoTKawamukaiM: Functional conservation of coenzyme Q biosynthetic genes among yeasts, plants, and humans. *PLoS One*.2014;9(6) : 10.1371/journal.pone.0099038 e99038 10.1371/journal.pone.0099038 24911838PMC4049637

# Peer review: Referee response for version 1

**DOI:** 10.21956/wellcomeopenres.10768.r17842

**Published:** 2016-11-25

**Authors:** Jim Karagiannis

**Affiliations:** 1 Department of Biology, University of Western Ontario, London, ON, Canada

The authors describe a genome-wide screen for fission yeast genes required for respiratory growth. While the study is similar to a previously reported screen by the same group ^1^ it differs in several important aspects:

1. A more up-to-date version of the gene deletion set is used.
2. An improved method for generating a prototrophic gene deletion library is employed.
3. Colonies are screened at lower densities allowing for more accurate measurements.

Taken together, these changes both improve the quality of the data set and provide novel information.

 The methods used for screening and data analysis are rigorous and clearly described.  All data have been deposited to the Open Science Foundation website and are freely available. The quality of the data set is supported by the fact that the identified genes are indeed enriched for those encoding mitochondrially localised proteins (as one would expect in a screen for respiratory deficient mutants). It should also be noted that many of the fission yeast hits in the screen have not been identified in budding yeast. I thus feel that the data represent an important, novel, and useful foundational resource for those with an interest in energy metabolism.

 The data also provide useful information with respect to various technical aspects of performing genome-wide screens. First, the authors - perhaps not surprisingly - show that the results of the screen are affected by the presence/absence of auxotrophic markers. Second, the study reveals large variability between biological repeats. While these results are somewhat troubling - in the sense that they make interpretation of this and other genome-wide screens more difficult - it is nevertheless critically important that researchers be made aware of:

1. Issues related to the confounding affects of auxotrophic markers.
2. The importance of performing multiple biological repeats when conducting genome-wide screens.

I have read this submission. I believe that I have an appropriate level of expertise to confirm that it is of an acceptable scientific standard.

## References

[1] MaleckiMBittonDRodriguez-LopezMRallisCGarcia CavaliaNSmithGBahlerJ: Functional and Regulatory Profiling of Energy Metabolism in Fission Yeast. *Preprints*.2016;10.1186/s13059-016-1101-2PMC512432227887640

# Peer review: Referee response for version 1

**DOI:** 10.21956/wellcomeopenres.10768.r17944

**Published:** 2016-11-24

**Authors:** Luis A. Rokeach

**Affiliations:** 1 Department of Biochemistry and Molecular Medicine, Université de Montréal, Montreal, QC, Canada

The title and abstract ****are appropriate.

 Article content: the results and design are adequately explained.

 The results presented by Malecki and Bähler constitute an interesting and needed contribution to the field energy metabolism. The techniques developed in this paper will be useful to other researchers wishing to carry out similar gene screenings.

 The data significantly broaden the knowledge acquired in a previous study. The wide variation in the biological datasets obtained with different repeats is intriguing. Some other potential explanations to these observations would have been useful. Certainly, these variations require further experimental scrutiny.

 It would have been very convenient that the genes identified in these screens be presented by their name (instead of number) on a lists appended to the article. As presented now, the data are less useful to the reader.

 As yeast mutants affected in respiration show also aging phenotypes, in future efforts it would be very interesting to investigate how the mutation identified would impact chronological life span.

I have read this submission. I believe that I have an appropriate level of expertise to confirm that it is of an acceptable scientific standard.

# Peer review: Referee response for version 1

**DOI:** 10.21956/wellcomeopenres.10768.r17607

**Published:** 2016-11-21

**Authors:** Christopher Herbert

**Affiliations:** 1 Institute of Integrative Biology of the Cell, CEA, CNRS, University Paris-Sud, University Paris-Saclay, Gif-sur-Yvette Cedex, France

The data note by Malecki and Bähler describes a screen to identify genes required for respiration in *S. pombe*. It differs from their previous study, in that an improved and more complete version of the deletion library was used, the method for transferring the deletions into a prototrophic background has been improved, colony sizes were determined at a much lower density and galactose medium was no longer used. Taken together, these changes should give a simpler and more robust dataset.

 The methods used to produce and analyze the dataset are clearly and succinctly explained and the treated and raw data are available in the Supplementary Tables.

 The authors find that, as they have previously shown, that approximately twice as many genes are identified in the auxotroph compared to the prototroph screen. This is no surprise, auxotrophic background is known to modify phenotypes, in particular *ura4* mutations often exacerbate respiratory phenotypes. Indeed, it could be argued that in future, resources would be better used if more biological repetitions were performed to produce a more robust dataset, rather than comparing auxotrophs with prototrophs.

 To my mind the most surprising, and perhaps worrying, result is the large differences seen between the biological repeats (Figure 1D) for both the prototroph and auxotroph strains. This is surprising as the “biological repeat” was essentially taking the strains out of the freezer, and they underwent two rounds of glucose growth before being tested for respiratory growth. This is an observation that requires further investigation; yeast biology would not be where it is today if strains had a 50% chance of changing their phenotype every time they came out of the freezer.

 The analysis in Figure 1D-F is interesting, I realize that this is a data note, not an article, but I have not been able to find the corresponding Tables it in the supplementary data. I recommend that the data Tables corresponding to Figure 1D-F be included in the supplementary data, and all the Tables should include standard gene names not just gene IDs, for most people interested in respiratory functions in *S. pombe *Cbp3 means something, SPCC4B3.17 does not.

I have read this submission. I believe that I have an appropriate level of expertise to confirm that it is of an acceptable scientific standard.
